# Arnold J. Levine and my career development

**DOI:** 10.1093/jmcb/mjz069

**Published:** 2019-08-16

**Authors:** Yigong Shi

**Affiliations:** Institute of Biology, Westlake Institute for Advanced Study, School of Life Sciences, Westlake University, Hangzhou 310024, China

I have known Arnold J. Levine, whom we fondly call Arnie, for over 22 years. Three important decisions in my career—job offer by Princeton University, the move from Princeton to Tsinghua, and professional development in China—were made with critical and helpful input from Arnie. I continue to count on him for advice on the growth of the newly founded Westlake University.

## Prelude

I was born and raised in China. Following undergraduate education at Tsinghua University in Beijing, I applied for graduate programs in the United States and began my experience at the Johns Hopkins University in 1990. I was enrolled in the Inter-campus Program in Molecular Biophysics and followed my thesis advisor Jeremy Berg to the east Baltimore campus where the School of Medicine was located. There I quickly became a big fun of Bert Vogelstein. At the time, Bert just proved p53 to be a tumor suppressor (Baker et al., 1989; Nigro et al., 1989), as opposed to an oncogene, and along with Ken Kinzler discovered the essential role of APC in familial adenomatous polyposis and most cases of colorectal cancers (Kinzler et al., 1991; Nishisho et al., 1991). On a sunny afternoon, Bert presented a public lecture on cancer in one of the hospital buildings and calmly reminded everyone that cancer is a genetic disease! This conclusion was rather startling to me and has had a lasting impact on my understanding of cancer. In that lecture, I learned a great deal about p53 and was introduced to the pioneering work by Arnold Levine (Linzer and Levine, 1979) and David Lane (Lane and Crawford, 1979).

As a result of Hopkins influence, I developed a strong interest in cancer research. In January 1996, I began my postdoctoral research in the Laboratory of Nikola Pavletich at the Memorial Sloan-Kettering Cancer Center (MSKCC). At the time, Nikola was collaborating with Arnie on the structural elucidation of the p53–MDM2 interactions (Kussie et al., 1996). With Nikola’s support, I began to explore the structural biology of BRCA1, cloned merely 13 months earlier (Futreal et al., 1994; Miki et al., 1994), and simultaneously launched a new project on the SMAD proteins in TGF-β signaling, which were just identified in fruit flies (Sekelsky et al., 1995), *Caenorhabtidis elegans* (Savage et al., 1996), and humans (Hahn et al., 1996). The SMAD project proceeded smoothly; by June 1996, I already identified and crystallized a C-terminal fragment (known as the MH2 domain) of the tumor suppressor SMAD4. SMAD4, also known as DPC4 (Hahn et al., 1996), was discovered by Scott Kern at the Hopkins Medical School.

## The Princeton job interview

With Nikola’s heavy input, I solved the X-ray structure of the SMAD4-MH2 domain in August 1996 and sent a manuscript off to *Nature* in September (Shi et al., 1997). One morning, Nikola shocked me by asking ‘Are you interested in a faculty position at Princeton University?’ He explained that Arnie had phoned to ask for a suitable structural biology faculty candidate in the Department of Molecular Biology (MolBio) at Princeton University. Attractive as the opportunity might have sounded, my initial response was a resounding NO, because I was completely unprepared for an independent research career. At the time, I had never thought about when to begin a real job hunt.

Two months later, as my first *Nature* paper was provisionally accepted, Nikola came back to me, ‘Arnie called again. Please send in your application, and it won’t hurt you.’ I still procrastinated for a few weeks but eventually sent in my application toward the end of January 1997. In addition to Jeremy Berg and Nikola Pavletich, I was quite fortunate to have the support of Joan Massagué, with whom I collaborated on the SMAD4 project, and Dinshaw Patel, then Head of the Structural Biology Division at MSKCC. A week later, Tom Silhavy, Chair of the Faculty Search Committee, invited me for an on-site interview.

In the morning of 27 February 1997, I showed up at Princeton for the interview. That was the first time in my life to visit this quiet college town, and I immediately fell in love with its picturesque environment and collegiate atmosphere. That was also the first and only real job interview in my entire life. I was arranged to have one-on-one interviews with 15 faculty members and felt quite nervous in the beginning. Shirley Tilghman actually quizzed me on the mechanism of imprinting that she just elaborated! I met Arnold Levine for the first time and had a pleasant conversation with him in his office. As a solicited candidate, I was expecting Arnie to give me some hints on the interview but heard nothing of this sort. The turning point was my carefully presented seminar—which was well received with a round of warm applause. After that, the atmosphere turned completely enjoyable and everything went more smoothly than I could possibly anticipate. Arnie, originally scheduled not to join the dinner, decided to come—a great sign, I thought. Tom Shenk, then Chair of the MolBio Department, also showed up at the dinner.

The dinner was held in Lahiere’s—one of Princeton’s premier French restaurants that opened in 1919 and was once Albert Einstein’s favorite restaurant (but was unfortunately closed for business in 2010). Before dinner, Arnie showed me an empty small table in Lahiere’s and commented, ‘Albert Einstein used to sit here. In a few years, if I invite you to dine at this table, you will soon receive tenure at Princeton*.*’ A portrait of Einstein was fixed to the wall right above the table. The dinner was completely relaxing and entertaining, with topics ranging from the early days of MolBio to hilarious anecdotes of faculty members. Arnie was a central source of humor and anecdotes, at times making me forget my role as a faculty candidate. Much to my surprise, both Arnie and Tom Shenk made repeated suggestions that I should start my independent research career at Princeton. Back to my hotel room at the Nassau Inn, I still felt a bit dizzy—were they making me an offer already? I remained sleepless throughout the night due to excessive excitement. One-on-one interviews continued the next day, and I fought hard to stay awake especially during the meeting with Eric Wieschaus right after lunch.

The good news did not arrive until one full month later, when Tom Silhavy called to make me an offer on 4 April 1997. I was so excited about the offer and accepted it right away on the phone. Apparently, Tom was unprepared for my immediate reply (despite its positive nature), hesitated for a few awkward seconds, and reminded me to negotiate a start-up package with Tom Shenk. I have been grateful to Arnie for his repeated invitations that ultimately landed me a most coveted job on earth.

My transition to Princeton was greatly smoothened in part because Arnie offered a postdoctoral position to my wife Renbin Zhao. Renbin was one year behind me in the Biology Department at Tsinghua and came to the US in 1991. After two school transfers, Renbin was eventually enrolled in the Hopkins BCMB Program. She joined the laboratory of Se-Jin Lee and defended her PhD dissertation in 1998. At the time of Renbin joining Arnie’s lab, microarray as a new technology was getting increasingly popular due to the embedded power of simultaneously analyzing the expression patterns of thousands of genes. In Arnie’s lab, Renbin carried out one of the first analyses of p53-regulated genes using oligonucleotide arrays (Zhao et al., 2000).

## Three pieces of priceless advice

Before I formally started at Princeton, I commuted between New York City and Princeton for a few months. During this period, I regularly went up to Arnie for advice and participated in the MolBio departmental retreat. As an upcoming Assistant Professor, I was a bit nervous about how to manage an independent laboratory. Arnie offered three pieces of priceless advice. The first piece of advice refers to how I should manage my time. ‘On the one hand, you will not succeed as a junior faculty member unless you treat yourself as a super postdoc in the lab for the first three to four years. You just have to work as hard as you can on your own research project and in the meantime train your students and postdocs to work on their projects. On the other hand, you will not be able to succeed fantastically as an established scientist if you are still a super postdoc after five years. You just have to begin to spend time communicating with your colleagues, writing grants, and managing projects in the lab.’

The second piece of advice relates to future research areas. ‘You can continue the research project that you have been doing as a postdoctoral fellow. It may even give you tenure; but it won’t make you a superstar. You will need to start an entirely new project that is independent of your doctoral or postdoctoral lab*.*’

The third piece of advice is on research grant preparation. External funding, especially that from the National Institutes of Health (NIH), is vital to every independent principal investigator. Similar to many other young principal investigators, I hesitated about when to submit my first NIH grant proposal. Most of my colleagues suggested ‘as early as possible’, reasoning that the first NIH grant was unlikely to be funded anyway and grant submission at an early date would help accumulation of relevant experience. Arnie disagreed with the majority, ‘Don’t waste your time submitting a grant with little data in it. Only prepare a grant when there are sufficient data, and there is no reason why the first research proposal cannot be funded.’

These three pieces of advice made perfect sense and have been dear to my heart. I wrapped up my research at MSKCC and formally joined Princeton in January 1998. I did exactly what Arnie suggested. I worked just like a super postdoc in the lab and managed my own research projects for the first four years. As soon as I arrived at Princeton, I expanded my research projects beyond TGF-β signaling into programmed cell death. After I determined the X-ray structure of the SMAD3-MH1 domain bound to DNA (Shi et al., 1998), I prepared my first NIH R-01 grant application in September 1998. This application received a rating of 8 percentile and was funded by the National Cancer Institute. Using the same strategy, I had an unusually high success rate with my NIH grant applications: 10 out of 11 were funded on the first submission. With ample research funds, I worked on research projects of my choice and quickly established my reputation as an independent structural biologist by 2001. By now, I have shared these three pieces of priceless advice with more than 100 members of my laboratory. Some of my past students and trainees, exemplified by Jijie Chai and Nieng Yan, have gone on to make lasting impact in their own research fields.

## China connection

Soon after I joined Princeton, Arnie left for New York City and became the President of the Rockefeller University. During the period of 1998–2002, Arnie put his heart and soul into the business of the Rockefeller University and became less accessible to me. Nonetheless, I had the opportunity of meeting him quite a few times, including twice in the President’s House at the Rockefeller University. Our topics often included China-related issues. Throughout the 1990s, China’s economic landscape was quite choppy. The inflation rate reached teens in the early 1990s, and the state-owned enterprises encountered many tough challenges. In the mid-to-late 1990s, the over-heated economy in China faced the disastrous possibility of hard landing. Scientific research in China was still quite primitive compared to the US. The prospects for the economy and scientific research appeared rather gloomy.

Contrary to the mainstream views, Arnie was unusually ardent about China’s future. ‘With so many Chinese studying in the US and excelling in various research fields, you can predict a bright future for China in 15 years.’ He believed in the tremendous value of the returnees in the modernization of China and went on to elaborate why China would rise in a way that would surprise most in the world. At the time, I was among the clear minority who were relatively optimistic about China’s future; but I still thought Arnie was too naively enthusiastic about China. The reality has proven Arnie to be outrageously insightful on the development of China. Even though I had already decided to come back to China since the inception of my postdoctoral training period, Arnie’s enthusiasm on China reinforced my decision.

Throughout my early years at Princeton, I had become increasingly appreciative of the ideas and advices that Arnie provided. I enjoyed a smooth career path at Princeton and was promoted to a tenured Full Professor in 2002–2003. Unfortunately, early success is often accompanied by premature anxiety. On the one hand, by 2003, the low-lying fruits in the research field of apoptosis had been picked. The leftover problems in this field were extremely challenging and had defied rigorous efforts from several research groups including mine. On the other hand, I began to pay undue attention to honorific titles and awards, in part motivated by the pleasant surprise of receiving the 2003 Irwin Sigal Young Investigator Award from the Protein Society. These circumstances engendered intense anxiety—perhaps a mid-life crisis—that I was ill-prepared to deal with. I again sought Arnie’s advice in his office at the Institute for Advanced Study. He appeared somewhat surprised by my confession. In the end, Arnie advised me to be patient and just focus on research and let the rest take care of itself with time. Although the advice fell short of my expectation, it turned out to be exactly how things had been developing for many years afterwards.

My first mid-life crisis was defeated in part through research expansion into another brand-new area—membrane protein structure and function. Starting in 2004, I assembled a team of graduate students and postdocs to work on intramembrane proteases—rhomboid, S2P, and γ-secretase. As this research effort was in full force, I received the invitation in May 2006 from the leadership of Tsinghua University for me to lead the life science community. After a brief consultation with Renbin, I accepted the job and began to make the transition immediately. It was an emotionally tough job for me to disassemble my Princeton lab that was simultaneously funded by five NIH R-01 grants. Most colleagues at Princeton were utterly surprised by my decision at a time when they believed I was rising rapidly in my career trajectory. Fred Hughson, a structural biologist and my next-door office neighbor, understood me from years of close interactions and graciously agreed to oversee my lab while I was away in China. Yibin Kang, a cancer biologist whom I helped recruit to Princeton just two years earlier, bid farewell to me barely after he settled down at Princeton.

Besides Fred and Yibin, Arnie was among just a few Princetonians who were unsurprised by my decision of resigning from the Warner–Lambert Parke–Davis Professorship. My departure was made more dramatic by the 2008 offer from the Howard Hughes Medical Institute (HHMI) as an Investigator. I traveled to Chevy Chase and tried, but unsuccessfully, to persuade HHMI to support my China effort. I had no choice but to decline the HHMI offer and joined Tsinghua in 2008. In many ways, I was merely fulfilling Arnie’s prediction about China. Rather than persuading me to stay on at Princeton, Arnie asked about how he could help me jump start my career in China. I told Arnie to wait for my call.

## From Tsinghua to Westlake

I was 23 when I left Shanghai Hongqiao Airport for San Francisco in April 1990. By the time I came back, I had been away from China for 18 years. The first major problem I encountered was the scientific culture that in many ways ran contrary to what I had been used to in the US. I fought what I believed to be the unhealthy aspects of the research culture and thankfully had Yi Rao always on my side. Yi resigned his endowed professorship from Northwestern University in July 2007 and became the Dean of the School of Life Sciences at Peking University. Yi and I teamed up in a few notable public appearances. One climax was an Editorial that we co-authored for *Science* magazine in the September 3, 2010 Issue (Shi and Rao, 2010), which immediately touched off a firestorm in China’s research landscape. To deal with the aftermath of this incident, I consulted a few Chinese colleagues and sought advice from Arnie and other friends in the US. Arnie encouraged me to stay calm and focus on research and institutional building.

**Figure 1:**
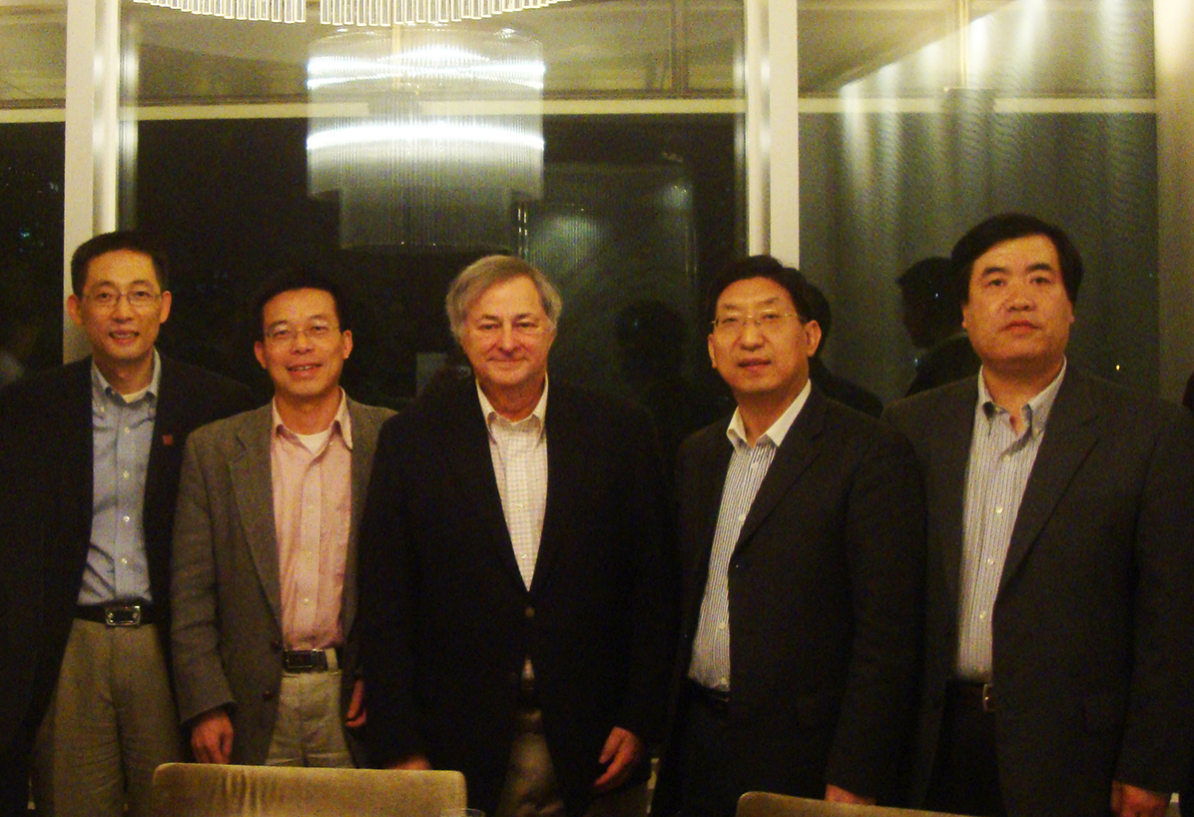
Arnie with some Chinese colleagues after giving a seminar at Tsinghua University on November 3, 2011. From left to right: Yigong Shi, Yi Zhang (a biochemist), Arnold Levine, Yixin Zeng (a cancer biologist), and Yuxin Yin (a former postdoc in Arnie’s lab).

I focused my energy on the Tsinghua task, which had been greatly aided by my experience and external connection accumulated during my 10-year tenure at Princeton. From 2008 to 2016, I sought help from many friends in the US, in particular Arnold Levine, Dinshaw Patel, and Steve Harrison. During this period, ~110 faculty members were hired to nearly quadruple the size of Tsinghua’s life science community. The Biology Department was upgraded to the School of Life Sciences, and the School of Pharmaceutical Sciences was founded. During this period, I invited Arnie three times to Tsinghua for research seminars and shared with him the progress (Figure 1). In addition to offering advices, Arnie always asked ‘What else can I do to help you and your institution?’

Despite China’s booming economy, the case for innovation is much less encouraging. The underpinnings of innovation are science and education. Having led the rapid rise of biomedical research at Tsinghua, I felt I had accomplished the task that was given to me in 2006. The time had come for me to do something entirely different. One idea was to promote science and education through the establishment of a brand-new university. This idea gained significant momentum after critical discussions among a group of returnees: this university should be small, research-oriented, world-class, and non-profit. After several years of preparation, we finally went into action. To cut a long story short, Westlake University—the first private research university in the history of the People’s Republic of China—was officially approved by the Chinese Ministry of Education in 2018. I was chosen by the Board of Trustees to be the Founding President. Different from all other universities in China, Westlake University is backed by both government support and private philanthropy and aims to attain a world-class echelon in research and education.

Arnie was among the first in the US to witness the process of founding the Westlake University. He again lent his generous support and graciously agreed to serve as a member of the Advisory Board of Westlake University. Just as Arnie predicted 20 years ago, the establishment of Westlake University is one of the many benefits that the returnees have brought to China.

## Retrospect

The year 2018 marks the 40th anniversary of China’s Reform and Opening-up Policy. The first batch of 52 students and scholars from mainland China arrived in the US on December 26, 1978, five days before the establishment of diplomatic relationship between the two nations. The legendary Chinese leader Deng Xiaoping made a historic visit to the US in January 1979, which was followed by decades of exchanges in education and research. By 2018, over 5 million Chinese students have studied in the Western hemisphere. The majority have returned to China, transforming the Chinese society in every aspect.

Together with Tom Shenk, Arnie made his first visit to China in 1993. During that trip, he met the assigned translator Mansuo Lu who was an undergraduate student in Wuhan University. A year later, with help from Arnie and Tom, Mansuo became one of the first batch of Chinese students in the MolBio graduate program. Joe Tsien and myself became the first Chinese faculty members in MolBio. I was in charge of foreign student admission into the graduate program and accepted ~30 Chinese students from 1998 to 2007. One of these students, Nieng Yan, is back to the MolBio Department as the first recipient of the Shirley Tilghman Professorship. In a way, Arnie and Tom spearheaded the Chinese flavor in the present multi-cultural environment of the MolBio Department.

My career development at several critical junctures had been positively influenced by Arnold Levine. Without Arnie’s insistence, I would not even go for the Princeton interview. With Arnie’s advice and support, I had a jump start at Princeton and made a decision of returning to China. Now, with Arnie in the Advisory Board along with many others, I and my colleagues hope to make Westlake University an innovative hub in research and education in the not-so-distant future—this is of course a considerably more challenging task than the previous ones I had accomplished. But a miracle is something that comes true when most people predict otherwise.
